# COGNITIVE VULNERABILITY TO TEMPERATURE BY REGION, HOUSING, AND NEIGHBORHOOD SAFETY IN HEALTH AND RETIREMENT SURVEY

**DOI:** 10.1093/geroni/igae098.0614

**Published:** 2024-12-31

**Authors:** Carina Gronlund, Erinn Cameron, Jinkook Lee, Jennifer Ailshire, Sara Adar

**Affiliations:** University of Michigan, Ann Arbor, Michigan, United States; Harvard T.H. Chan School of Public Health, Boston, Massachusetts, United States; University of Southern California, Los Angeles, California, United States; University of Southern California, Los Angeles, California, United States; University of Michigan, Ann Arbor, Michigan, United States

## Abstract

With a changing climate, understanding how temperature affects the cognitive function of older adults is critical. Extreme temperatures might influence cognitive function via multiple physiological processes and may be stronger in certain spaces, including poor-quality housing and unsafe neighborhoods where residents may not open windows. Using biennial surveys from ~40,000 respondents from 1995-2020, we characterized associations between total cognition scores (35-point scale) and temperatures overall and by census region, housing condition (poor-to-excellent), and neighborhood safety (feel safe walking after dark scale 1-7). We operationalized temperatures as monthly regional means of daily minimum temperatures from NOAA monitors 1-12 months prior to the surveys, converted to region-specific percentiles. We regressed cognition on nonlinear temperature lags, controlling for individual (fixed slope), precipitation, season, survey wave, and age. We then interacted home condition and neighborhood safety with temperature, controlling for self-efficacy, functional limitations (ADLs), and comorbidities. Overall, we found lower cognitive performance with colder temperatures 6 months prior (-0.1 points, 95% Confidence Interval: -0.2, 0.0 for the 1st vs. 50th percentile). For hot temperatures, results were regionally heterogeneous. We saw lower cognition with higher temperatures in some regions (e.g., in the East North Central region, 1.5 (95% CI: -2.2, -0.4) and 3 (95% CI: -1.6, -0.5) points lower at 99th vs. 50th percentile 1 and 3 months prior, respectively). Hot temperature associations were worse in unsafe neighborhoods but not by home condition. This suggests that climate adaptation programs, including safe cooling spaces, might benefit cognitive health.

